# An intervention to improve paediatric and newborn care in Kenyan district hospitals: Understanding the context

**DOI:** 10.1186/1748-5908-4-42

**Published:** 2009-07-23

**Authors:** Mike English, Stephen Ntoburi, John Wagai, Patrick Mbindyo, Newton Opiyo, Philip Ayieko, Charles Opondo, Santau Migiro, Annah Wamae, Grace Irimu

**Affiliations:** 1 KEMRI Centre for Geographic Medicine Research – Coast, & Wellcome Trust Research Programme, P.O. Box 43640, Nairobi, Kenya; 2 Department of Paediatrics, University of Oxford, Oxford, UK; 3 Division of Child Health, Ministry of Public Health and Sanitation, Nairobi, Kenya; 4 Department of Paediatrics, College of Health Sciences, University of Nairobi, Nairobi, Kenya

## Abstract

**Background:**

It is increasingly appreciated that the interpretation of health systems research studies is greatly facilitated by detailed descriptions of study context and the process of intervention. We have undertaken an 18-month hospital-based intervention study in Kenya aiming to improve care for admitted children and newborn infants. Here we describe the baseline characteristics of the eight hospitals as environments receiving the intervention, as well as the general and local health system context and its evolution over the 18 months.

**Methods:**

Hospital characteristics were assessed using previously developed tools assessing the broad structure, process, and outcome of health service provision for children and newborns. Major health system or policy developments over the period of the intervention at a national level were documented prospectively by monitoring government policy announcements, the media, and through informal contacts with policy makers. At the hospital level, a structured, open questionnaire was used in face-to-face meetings with senior hospital staff every six months to identify major local developments that might influence implementation. These data provide an essential background for those seeking to understand the generalisability of reports describing the intervention's effects, and whether the intervention plausibly resulted in these effects.

**Results:**

Hospitals had only modest capacity, in terms of infrastructure, equipment, supplies, and human resources available to provide high-quality care at baseline. For example, hospitals were lacking between 30 to 56% of items considered necessary for the provision of care to the seriously ill child or newborn. An increase in spending on hospital renovations, attempts to introduce performance contracts for health workers, and post-election violence were recorded as examples of national level factors that might influence implementation success generally. Examples of factors that might influence success locally included frequent and sometimes numerous staff changes, movements of senior departmental or administrative staff, and the presence of local 'donor' partners with alternative priorities.

**Conclusion:**

The effectiveness of interventions delivered at hospital level over periods realistically required to achieve change may be influenced by a wide variety of factors at national and local levels. We have demonstrated how dynamic such contexts are, and therefore the need to consider context when interpreting an intervention's effectiveness.

## Introduction

The poor quality of care offered to children in hospital in many low-income settings [1,2], including Kenya [3,4], has been widely reported. The challenge is now therefore to define interventions that might improve this care. We have previously described the design of a randomized, parallel group intervention study that aims to investigate whether a package of interventions delivered to Kenyan government district hospitals can improve paediatric and newborn care [5]. Similarly, we have described the development and content of a major part of the intervention package: evidence-based clinical practice guideline booklets (CPGs), a standard paediatric admission record form (PAR) [6] and a five-day training course focusing on emergency and admission care and use of the CPGs (Emergency Triage Assessment and Treatment plus Admission Care, ETAT+) [7]. Additional aspects of the intended intervention package included: external support supervision, local facilitation, performance assessment, and feedback.

However, training and guidelines may only result in changes in the provision of care in settings with adequate physical and human resources. Supervision and feedback may have little effect if staff and management are preoccupied with other priorities, while a specific intervention effect might be hidden by any broad, major health sector developments. At a local level, the intervention delivered may work to different degrees in hospitals of different sizes or those that lose key personnel or trained staff. Here, therefore, we describe the hospitals and the health system as contexts within which this multi-faceted intervention was delivered. Understanding the dynamic nature of this context and its potential for influencing the effect of the intervention is an important precursor to understanding or generalizing any results [8,9]. This report also provides the backdrop to additional specific and prospectively specified studies examining health worker motivation [10], the barriers that might prevent health workers following guidelines [11], and the perspectives of the research team and the recipient health workers on the adequacy of delivery of the intervention [12] (see Appendix 1).

## Methods

### The Kenyan health sector

Kenya is a low-income country with a population of 33 million and a GDP per capita of $580 in 2006 USD . In 2004 and 2005, government spending on health was $9.1 per capita representing 7.7% of total government spending . The level of government spending on health has been increasing in absolute terms since 2001, although remaining relatively stable as a proportion of total government spending  while general economic growth improved from 0.4% to more than 6% over the period from 2000 to 2006 . Although the country made important health gains in the decades leading up to 1990, this was followed by a period of stagnation and then deterioration, at least for child survival, with mortality of children less than 5 years old increasing from 97 per 1000 in 1990 to 121 per 1000 in 2003 . Among many factors that will have contributed to these worsening health indicators, economic decline, a public sector employment freeze, and, until recently, minimal investment in health services despite continued population growth and an emerging human immunodeficiency virus (HIV) epidemic are perhaps the most important.

Organisationally, publicly provided health services are based around the district administrative level. Districts in turn are responsible to provincial (regional) and then national offices. Each district is normally served by one designated district hospital. The district hospitals are run by a hospital management team, usually comprised of a senior clinician, a senior nurse, a pharmacist, an administrator, and other heads of department. This team is responsible to a local hospital management board. The district hospital often provides primary and inpatient care services to a surrounding urban and nearby rural catchment area and, in principle, also provides referral care and inpatient services in support of a network of rural primary care facilities spanning the district. At the time of study design Kenya had 70 districts. These were subsequently divided to yield a total of 140 districts in 2007, mid-way through the intervention study, although for practical purposes this did not impact on the study. It is not possible to summarise adequately the entire scope of the health policy context, however, in principle government providers were expected to supply free health care services for children less than five years of age.

### Hospital selection and data collection

**Figure 1:**
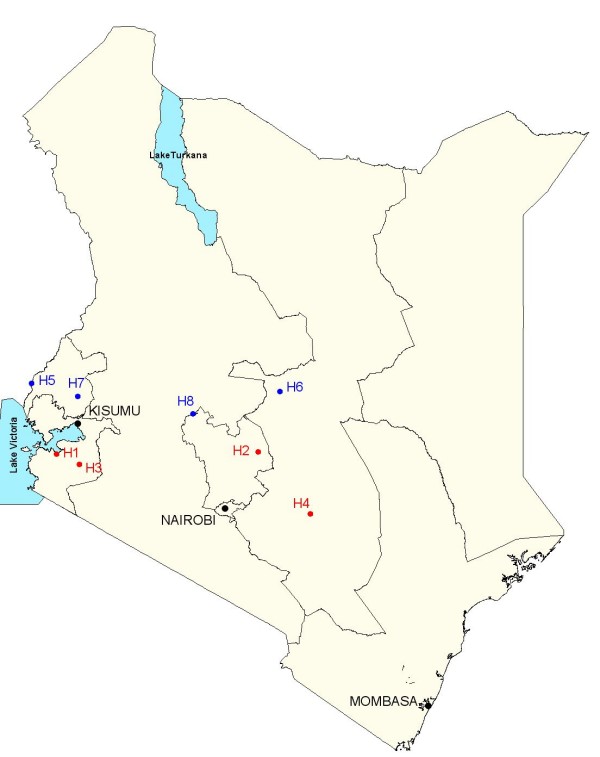
**Map of Kenya showing location of intervention (H1 to 4) and control (H5 to 8) hospitals**.

In total, there are thought to be over 300 hospitals providing general inpatient services in Kenya [13]. Of these, just over 120 are operated by the government, while faith-based or not-for-profit organizations support a similar number. The rationale for selection of the eight study hospitals and their 'recruitment' has been explained elsewhere [5], while their location is indicated in Figure 1. It is clearly hard to claim that only eight facilities are a true, nationally representative sample. We therefore aimed to document and describe key health system attributes, related to care of the severely ill newborn or child, that would allow others to consider how representative this sample is of the wider Kenyan or regional hospital sector Surveys were conducted by three teams of four or five health workers specifically trained for the task and led by at least one full-time member of the research team. Data were collected using multiple tools, adapted from previous work [4], that aimed to describe hospital care within the classical Donabedian framework of structure, process, and outcome [14]. Relevant tools are briefly described in Table 1. Although the specific structure attributes would be linked to those of process and both to specific outcomes in the classical health production model, this precise linear thinking is rarely possible when attempting a hospital-wide quality of care assessment such as the one described. Instead, broad panels of indicators have been assessed that help define quality of structure, quality of process, and quality of outcomes as discrete although linked phenomena.

**Table 1:** Simple description of tools used and purpose of each tool

***Data collection Tool***	***Focus of data collection***
Facility inventory checklists. Data based on the observation of the survey team leader and, where necessary, the response of senior administrators or department heads.	To record availability of water/electricity. To document staff numbers and department allocations. To record the presence/absence of key items of equipment, essential drugs, essential consumables, and availability of laboratory tests. For equipment or laboratory tests, the item had to be functional as well as present. To record aspects of the organization of services – for example whether or not triage was operational in clinic areas providing walk-in services for sick newborns and children.
Medical record data abstraction tool	To record what was documented for newborns and children about the admission clinical assessment, diagnosis, and treatment, key aspects of inpatient care and outcome. Aim: 400 randomly selected case records per site from the six months prior to the survey.
Caretaker interview (used after gaining informed consent)	Structured interview questionnaire including specific data collected at patient discharge on caretaker's knowledge of the patient's diagnosis and post-discharge treatment. Aim: 50 consecutive, prospectively identified admissions during the survey period.

**Major events/changes structured interview**	For post-baseline surveys a short structured interview with senior hospital staff was performed to identify major new initiatives affecting the hospital or major staff movements.

While data on structure (facility inventory) and from caretaker responses represent point-in-time, actual observation that collected from medical records and from structured interviews is retrospective in nature and potentially more subject to bias. While it is unlikely that major events affecting hospitals would be misreported during interviews, the quality of information for less major events, such as the details of staff rotation, may be affected. Data collected from medical records suffers from the problem that it is assumed what is not recorded is not done. For patient assessment tasks this is particularly the case, and such process indicators reflect both quality of documentation and practice. However, the assessment indicators selected are fundamental to appropriate care for sick children with common conditions (for example, the child's weight) and were part of existing standards of practice in the form of disease-specific government practice guidelines. Further process indicators, based on correctness of drug or fluid prescription for example, are less subject to such biases. Using these tools, the descriptions presented of the structure, process, and outcome characteristics of the hospitals as contexts are based on comprehensive surveys conducted for two weeks at each site between 9 July and 19 August 2006 prior to any intervention (Survey 1). We planned to repeat surveys in all sites at approximately five to six months (Survey 2), 11–12 months (Survey 3), and 17–18 months (Survey 4) after randomly allocating hospitals to two groups of four (referred to as intervention and control hospitals, see below and [5]) and after initiation of the intervention. Data on national and local policy and management changes collected during these follow-up surveys are presented, but data describing structure, process, and outcomes of care will be presented elsewhere.

For surveys, initial training was conducted for all staff over two weeks and was based around a 'Survey Workers Handbook' that described the study, approaches to data collection, and the specific rules for recording data related to every question for each tool. Practical training included: thorough familiarization with the study purpose; relevant communication skills including obtaining informed consent; discussion of bias and the importance of objectivity among survey staff; question by question discussion of each survey tool to develop a common understanding and agreed rules for data recording; role play or classroom practice for data collection with each tool and three days of practical experience in data collection at the National Hospital. Group discussion was used to resolve remaining uncertainties over data recording with all final decisions recorded in an updated and final version of the 'Survey Workers Handbook' carried by each survey member.

### Documenting change in the hospitals as contexts

At the outset of this programme, we established a basic approach to record, prospectively, major health system events beyond the scope of our intervention, relevant to child and newborn health, that might influence health sector performance. This involved monitoring the passage of any parliamentary bills, directives from the Minister of Health or Finance or key senior civil servants in these ministries, and monitoring the countries two major newspapers. In addition, data were collected using structured interviews with hospital staff (see Table 1) and cross-checked during contact with facilitators. Relevant findings from these activities, organized with respect to the conduct of hospital surveys, are presented together with a brief overview of the Kenyan health sector collated from published data or reports.

### Experience and results

#### Study hospitals

Key characteristics of the study hospitals at baseline are illustrated in Additional File 1 and their location in Figure 1.

#### Structure and service organisation

Study hospitals had generators and for the main part were able to maintain electricity supplies but in four hospitals (three intervention, one control) considerable problems with water supply were present. Acute, walk-in care for sick children under five years of age is generally provided as part of maternal and child health clinics during the working week, and by general outpatient or casualty departments at nights and weekends. In five hospitals (three intervention, two control) clinical officer interns, and in two hospitals medical officer interns (one intervention, one control), were part of the clinical workforce providing admission paediatric care. Both cadres of intern rotate for three months through the paediatric department, and although they considerably increase the total number of clinical service providers the result is a rapidly changing clinical workforce. Nursing numbers are low, with one nurse for each 12 to 18 paediatric beds even during the day. At night in smaller hospitals (hospitals H1, H4 and H5) there was often only one nurse on duty on the paediatric ward, and even during the day it was rare for a nurse to be specifically assigned to the newborn nursery.

Hospitals were relatively poorly equipped to deal with a seriously ill child. Based on a set of priority items for providing care that might have to be used to manage a paediatric (three areas) or neonatal emergency (one area), hospitals were lacking between 27% and 55% of items. Maternal and child health clinics and outpatient/casualty departments were most deficient. The proportion of drugs named in the CPGs as essential to admission or early care (n = 29) missing from either of the paediatric ward or the pharmacy varied from 41% to 62% although, importantly, all of these drugs were available in at least one of these locations at the time of the survey in all eight hospitals.

#### Process of care

Medical records documenting the admission event for infants and children aged 7 days to 59 months were written as short, non-standardised, free-text notes at all eight sites. Retrieval of archived records was possible in seven sites, but in one control site (H5) large numbers of patient records were missing. This was attributed to a lack of stationery and therefore use of a patient-held outpatient book (retained by the caretaker on discharge) even for inpatient documentation. In records, examined age was generally well-documented for inpatient children, but weight was recorded in fewer than 50% of admissions in seven of eight hospitals, while temperature and vaccination status were documented in fewer than 10% of admitted children in six of eight sites. Documentation of specific clinical signs important for the diagnosis and severity classification of common illnesses was very poor (Additional File 1) with the exception of pallor and, occasionally, cyanosis. Across all eight sites, even in the face of poor documentation, 347 children had recorded clinical signs indicating a probable need for lumbar puncture (LP) according to CPG criteria. Only nine LPs were documented as performed. In terms of management, quinine loading doses were prescribed to fewer than 10% of admitted malaria cases for whom the drug was used in seven of eight sites. Only 9 of 238 (2.5%) children admitted for intravenous fluid therapy for severe dehydration had a fluid prescription indicating the correct volume of fluid and duration of administration. In 122 cases of children admitted with severe malnutrition, a prescribed feeding plan was available for only nine (7%).

### Outcomes

Among study hospitals the number of paediatric admissions varied from just under 1,000 to nearly 5,000 per year with inpatient paediatric mortality varying from 5.2% to 13.7%. Outcome indicators reflecting caretakers' knowledge about their child's illness and management at discharge showed that more than 50% of caretakers knew their child's diagnosis in six of eight sites. However, in only two hospitals were 50% or more of caretakers aware of the frequency with which they had to administer discharge drugs.

### Context

In Additional File 2 we outline changes in the health sector originating at the national level that might influence hospital or health worker performance in all eight hospitals. Across the eight hospitals, income generated from cost recovery (user fees) and available to the hospital management team to spend, varied considerably from approximately $10,000 per month to approximately $45,000 per month (Additional File 3). This variation reflects both variability in hospital size and the ability of the catchment population to pay. Although, in theory, services for children are free, in practice all hospitals levy a bed charge on the caretaker staying with the child and payment is often required for specific treatments or investigations. In three hospitals (H1, H3 and H5), there were major changes (>50% increase) in bed-day charges over the 18 months of the intervention. Although all government hospitals, each site had at least one additional partner, varying from non-governmental organizations to bilateral aid programs, providing direct local support (Additional File 3). In seven of eight sites, partners were helping support provision of HIV services. None of the hospitals were receiving broad support from partners for provision of newborn or child health services, although in one hospital at baseline (H6) and a second during the intervention (H1) ready-to-use nutritional products for severe malnutrition were provided. During the 18 months of the study, both the government and local partners provided inputs that may have helped improve hospital care for newborns and children. These inputs varied and included, for example: funds for maintenance and renovation of facilities; improved drug and consumable supplies from the Kenyan Medical Supplies Agency (KEMSA); provision of oxygen concentrators and newborn incubators; and construction of boreholes to improve water supplies (Additional File 3).

At least as significantly, regular changes in senior staff were observed at all sites. Examples include two changes within 18 months of the medical superintendent (hospitals H5 and H6), four changes within 18 months of senior nursing personnel in paediatric areas (H3), and major internal rotations (≥ 20 staff) with exchange of nurses familiar with the intervention for those with none (H1, H2 and H3). Although there were new postings of medical and nursing staff, these just kept pace with transfers and resignations to maintain total staffing numbers reasonably constant, although for some smaller hospitals (H4) numbers of general medical officers varied considerably and meant for prolonged periods none was allocated to the paediatric wards. The variability in clinical medical staffing was particularly pronounced in the two largest hospitals (H3 and H7) that received medical officer interns where the intervention of 18 months spanned six scheduled changeovers in medical personnel in these sites.

## Discussion

The study design aimed to balance intervention and control group hospitals on the basis of size, presence of a paediatrician and medical officer interns, and some basic characteristics of the geographic location [5] (Additional File 1, Figure 1). It can be seen this also resulted in reasonable balance with respect to many gross structural attributes of hospital care, including organizational aspects of care, availability of human resources, equipment, and drugs at baseline. Intervention group hospitals, however, tended to have higher inpatient paediatric mortality. For indicators related to the process of hospital care, intervention group hospitals and control group hospitals fared equally badly in general at baseline. These baseline data also indicate that little progress had been made in improving paediatric care, or in implementing available WHO and national treatment recommendations in the four years between the baseline surveys reported and similar surveys in 2002 involving seven of these hospitals [3,4].

Although the baseline cross-sectional data provide some reassurance that the process of randomization helped achieve group balance, the highly dynamic reality of hospitals evidenced by the prospectively organized approach to description underscores the need for caution when interpreting the results of the intervention in the future. The data presented are, we hope, an aide to those interested to consider for themselves the plausibility of any cause and effect relationship attributed to the intervention. National level developments, such as improved health spending or introduction of new management approaches, both of which occurred during the intervention period, would be expected to affect all hospitals in a similar way and no specific regional initiatives were encountered. However, we cannot discount the possibility that national directives are differentially applied and/or significantly affected by a hospital's local administration and management, potentially affecting uptake of new hospital and health worker practices. More obviously, it is clear that hospitals work with a range of partners and initiatives at a local level. None during our observations targeted improvements in child and newborn health care broadly other than the planned intervention. However, some may have influenced the quality of service provision for specific aspects of care, such as the provision of incubators for the newborn, effects that might be attributed to our broad intervention unless documented. Alternatively, the intervention's effectiveness might be negatively affected by prioritization of other areas, and in this regard it is interesting to note that seven of eight hospitals were working with non-governmental partners supporting HIV-related activities often bringing considerable resources.

Such rich contextual data have a number of implications. Firstly, the diversity encompassed by the simple term 'hospital', even in a sample of only eight in one low-income setting, is striking. This is rarely considered in national or international debate, or in interpretation of results of research or evaluation. Secondly, hospital management and staffing are clearly likely to be poorly described by a single round of data collection. In the light of our study, it should also be clear, despite some reassurance provided by randomization, that there is considerable scope for residual confounding and bias to influence the direction of results, both of which may be time-varying in direction or magnitude. Such careful descriptions of the type we have attempted may allow the plausibility of any causal relationship between intervention and response to be scrutinized and debated, but do not overcome these potential problems of bias and hidden confounding. While very large randomized controlled trials might be expected to provide a solution, it is questionable whether they are feasible and even if performed it would appear prudent that they still be accompanied by detailed description.

Perhaps the most striking finding resulting from our attempts to track changes in hospital contexts is the rapidity of turnover in senior hospital management, senior departmental nursing staff, and clinical service providers relevant to delivery of paediatric and newborn services. Such turnover was apparent in all hospitals and resulted from staff transfers between hospitals, locally controlled internal staff rotation of nurses, scheduled rotation of clinical staff linked to training requirements and, where clinical staff were few, reallocation of clinical staff away from paediatric and newborn areas that were considered a low priority. Thus any intervention aimed at changing service provision must transcend these staff dynamics to be successful in changing practice over the long term. A factor that encouraged us to explore the role of a local facilitator as part of the intervention, alternatively, or in addition, hospitals or implementers interested in achieving long-term change may need to develop strategies for expert staff retention. While this might encompass incentives to retain staff in rural or underserved areas, thought should also be given to revising routine staff rotation policies.

## Conclusion

We have presented a detailed description of a set of Kenyan government rural hospitals included in an intervention study examining an approach to improving paediatric and newborn care. We have attempted to characterize important aspects of the national setting, the hospitals, and the major changes at national and local levels that might affect the results of an intervention delivered over an 18-month period. Such data are thought to be essential to understanding and generalizing the results of public health efficacy or health systems intervention studies of this kind where interpretation is based largely on the plausibility of linking interventions to outcomes [9]. It is clear that hospitals as contexts are highly dynamic. Among the national level changes we documented, including the post-election violence in Kenya, we did not identify any that might obviously influence the performance of any one or any subset of hospitals. At the local level, major changes in all hospitals in senior personnel and clinical and nursing staff would seem the most likely general threat to the long-term success of any intervention. It is also possible that key local personnel changes or the actions of local partners could have a major influence on the success of interventions aiming to change the provision of services, reinforcing the case for as detailed a description as possible of the context and process of intervention when interpreting the outcomes of health system interventions. The tools we have developed and used provide one way to capture appropriate data. Such tools could be further adapted for health system-wide assessments examining the quality of hospital care at a national level. These data could inform key policy developments and help target resource delivery in line with service provision and equity goals.

## Competing interests

The authors declare that they have no competing interests.

## Authors' contributions

The idea for the study was conceived by ME who obtained the funding for this project. Preparation for and conduct of the study was undertaken by all authors. ME produced the draft manuscript to which all authors contributed during its development. All authors approved the final version of the report.

## Appendix 1

Summary of linked research studies intended to facilitate interpretation and appraisal of the final results of a multifaceted, hospital care improvement intervention in Kenyan rural, government hospitals.

***Study 1***: An intervention to improve paediatric and newborn care in Kenyan district hospitals: Understanding the context – *Intended to describe relevant health policy and institutional environment and changes over the 18 months intervention and study hospitals in terms of human and material resource capacity and indicate nature (quality) of care provided for children and newborns at baseline*.

***Study 2 ***Contextual influences on health worker motivation in district hospitals in Kenya – *Intended to explore health worker motivation in study hospitals prior to any intervention as motivation is considered to be a potentially important modifier of implementation success*.

***Study 3***: Documenting the experiences of health workers expected to implement guidelines during an intervention study in Kenyan hospitals – *Intended to describe from the health workers' perspective factors that may prevent broad use of the guidelines with data collection undertaken 4–5 months after initiating the intervention*.

***Study 4***: Implementation experience during an eighteen month intervention to improve paediatric and newborn care in Kenyan district hospitals – *Intended to describe how the intervention was actually delivered over the 18 months and explore health workers views of different intervention approaches after 16–18 months of intervention*.

These studies are aimed at allowing others to consider: i) How this Kenyan setting might be representative of their own setting, facilitating an assessment of generalisabilty, ii) How well the intervention was delivered, its 'adequacy', and, iii) The range and complexity of factors that might influence success or failure of the intervention as they assess the plausibility of links between intervention and reported results.

## Supplementary Material

Additional file 1 **Table S2. Basic workload statistics, structural, process and outcome indicators relevant to paediatric and newborn care in all hospitals at baseline (hospitals H1 – H4 later received the full intervention, H5 – H8 acted as contemporaneous controls)**. The data provided provides a description of the hospitals at baseline and the findings of the baseline quality of care surveys. Click here for file

Additional file 2 **Table S3. National level contextual factors potentially influencing effectiveness of the hospital based intervention programme to improve quality of paediatric and newborn care**. The data provided indicate how the national health policy context changed during the progress of the study. Click here for file

Additional file 3 **Table S4. Hospital level contextual factors potentially influencing effectiveness of the hospital based intervention programme to improve quality of paediatric and newborn care**. The data provided indicate how the local health policy and organizational context changed during the progress of the study. Click here for file
