# Supplementary material for: An intervention to improve paediatric and newborn care in Kenyan district hospitals: Understanding the context
Source: Implement Sci. 2009 Jul 23;4:42. doi: 10.1186/1748-5908-4-42 (PMC2724481; doi:10.1186/1748-5908-4-42)
Supplement: Additional file 1 — Table S2. Basic workload statistics, structural, process and outcome indicators relevant to paediatric and newborn care in all hospitals at baseline (hospitals H1 – H4 later received the full intervention, H5 – H8 acted as contemporaneous controls). The data provided provides a description of the hospitals at baseline and the findings of the baseline quality of care surveys. [file 1748-5908-4-42-S1.doc]

**Table 2**. Basic workload statistics, structural, process and outcome indicators relevant to paediatric and newborn care in all hospitals at baseline (hospitals H1 – H4 later received the full intervention, H5 - H8 acted as contemporaneous controls)

|  | **Hospitals** | | | | | | | |
| --- | --- | --- | --- | --- | --- | --- | --- | --- |
|  | **H1** | **H2** | **H3** | **H4** | **H5** | **H6** | **H7** | **H8** |
| **Workload statistics** |  |  |  |  |  |  |  |  |
| Annual paediatric admissions | 2356 | 3160 | 4205 | 996 | 2925 | 1058 | 4738 | 2128 |
| All cause paediatric ward mortality rate | 13.7% | 5.4% | 7.3% | 8.0% | 6.5% | 5.2% | 4.1% | 7.3% |
| Annual deliveries on maternity unit | 1750 | 4951 | 7500 | 2080 | 1697 | 1799 | 4235 | 3595 |
| **Structure Indicators** |  |  |  |  |  |  |  |  |
| Significant water supply problem | Y | N | Y | Y | Y | N | N | N |
| Paediatric Beds | 32 | 50 | 55 | 32 | 34 | 35 | 65 | 40 |
| Newborn nursery cots | 8 | 13 | 9 | 4 | 6 | 6 | 14 | 11 |
| Consultant Specialists: (Paediatricians) | 3(0) | 3(0) | 5(1) | 2(0) | 2(0) | 1(0) | 5(1) | 2(0) |
| General Medical Officers in hospital | 2 | 4 | 5 | 4 | 4 | 4 | 5 | 4 |
| Site for Medical Officer Intern training | N | N | Y | N | N | N | Y | N |
| Clinical Officers in hospital | 15 | 21 | 29 | 19 | 18 | 18 | 27 | 20 |
| Site for Clinical Officer Intern training | Y | N | Y | N | N | N | Y | Y |
| Nurses in hospital (all cadres) | 140 | 161 | 207 | 120 | 114 | 128 | 284 | 144 |
| Nurses per day shift, Paediatric Ward | 2 | 4 | 3 | 2 | 2 | 3 | 4 | 3 |
| Nurse per bed ratio, daytime on paediatric ward | 16.0 | 12.5 | 18.3 | 16.0 | 17.0 | 11.7 | 16.3 | 13.3 |
| Nurses specifically allocated to newborn nursery (day) | 0 | 0 | 1 | 0 | 0 | 0 | 2 | 1 |
| Emergency equipment from four sites* - % missing | 42% | 32% | 56% | 56% | 36% | 32% | 28% | 30% |
| Essential Drugs**: % missing either ward or pharmacy | 59% | 48% | 45% | 48% | 41% | 55% | 62% | 41% |
|  |  |  |  |  |  |  |  |  |
| **Process Indicators** |  |  |  |  |  |  |  |  |
| Number of medical records evaluated | 395 | 400 | 398 | 399 | 162 | 401 | 400 | 399 |
| Age of child documented | 95% | 99% | 93% | 97% | 91% | 98% | 100% | 100% |
| Weight documented | 41% | 33% | 91% | 64% | 48% | 4% | 32% | 2% |
| Temperature documented | 3% | 11% | 5% | 26% | 2% | 0% | 8% | 2% |
| Vaccination status documented | 7% | 7% | 4% | 5% | 0% | 7% | 65% | 32% |

|  | **H1** | **H2** | **H3** | **H4** | **H5** | **H6** | **H7** | **H8** |
| --- | --- | --- | --- | --- | --- | --- | --- | --- |
| ***Malaria specific indicators*** |  |  |  |  |  |  |  |  |
| Number of cases with a malaria diagnosis | 328 | 262 | 244 | 290 | 131 | 244 | 352 | 164 |
| Conscious level documented | 1% | 1% | 0% | 0% | 1% | 0% | 9% | 0% |
| Acidotic or deep breathing documented | 0.30% | 0% | 0% | 0% | 0% | 0% | 0% | 0% |
| Degree of Pallor documented | 75% | 78% | 94% | 86% | 63% | 90% | 91% | 64% |
| Cases with a disease severity classification | 24% | 9% | 6% | 7% | 6% | 2% | 3% | 26% |
| Quinine loading dose prescribed | 3% | 2% | 5% | 3% | 5% | 25% | 1% | 0% |
| ***Pneumonia specific indicators*** |  |  |  |  |  |  |  |  |
| Number of cases with a pneumonia diagnosis | 116 | 178 | 145 | 149 | 20 | 170 | 95 | 178 |
| Respiratory rate documented | 6% | 0%(0) | 7% | 2% | 0% | 0% | 9% | 1% |
| Indrawing documented | 19% | 13% | 11% | 11% | 5% | 5% | 40% | 11% |
| Cyanosis documented | 2% | 31% | 52% | 30% | 15% | 2% | 39% | 22% |
| Cases with a disease severity classification | 16% | 13% | 10% | 5% | 20% | 5% | 18% | 7% |
| ***Diarrhoea / dehydration specific indicators*** |  |  |  |  |  |  |  |  |
| Number of cases with a dehydration diagnosis | 92 | 46 | 111 | 71 | 14 | 100 | 87 | 136 |
| Sunken Eyes documented | 14% | 11% | 8% | 1% | 0% | 3% | 10% | 5% |
| Skin turgor / skin pinch documented | 18% | 4% | 6% | 0% | 0% | 21% | 2% | 0% |
| Cases with severity of dehydration classified | 57% | 60% | 61% | 57% | 36% | 59% | 30% | 46% |
| Cases with correct fluid prescription (volume and time) | 0% | 0%(0) | 19% | 0% | 0% | 15% | 10% | 0% |
|  |  |  |  |  |  |  |  |  |
| **Outcome Indicators (from 50 caretaker interviews)** |  |  |  |  |  |  |  |  |
| Caretakers knowing diagnosis at discharge | 87% | 28% | 82% | 83% | 78% | 76% | 85% | 82% |
| Caretakers knowing frequency of discharge drugs administration | 34% | 6% | 22% | 59% | 16% | 31% | 5% | 50% |
| Median user fee charged (KSH) | 120 | 600 | 210 | 150 | 120 | 200 | 400 | 710 |

*Oxygen delivery system, Inhaled therapy for severe asthma (not nursery), Suction device, Diazepam (not nursery), Bag valve mask device, NG tubes, Fluids for shock, iv giving sets and cannulae, Syringes and needles, 50% Dextrose, Adrenaline for injection (not nursery), Weighing scales, Resuscitation couch (not nursery), Warm space for neonatal resuscitation (Nursery only), Phototherapy equipment (Nursery only)

**Adrenaline, Aminophylline, Amoxycillin, Benzyl Penicillin, Ceftriaxone, Chloramphenicol, Co-trimoxazole, Diazepam, Gentamicin, Glucose 50%, Iron, Mebendazole, Metronidazole, Multivitamins, Nalidixic acid, Nystatin, Oral Rehydration Solution, Paracetamol, Phenobarbitone, Prednisolone, Quinine, Salbutamol, Tetracycline Eye Ointment, Vitamin A, Vitamin K
