# Supplementary material for: An intervention to improve paediatric and newborn care in Kenyan district hospitals: Understanding the context
Source: Implement Sci. 2009 Jul 23;4:42. doi: 10.1186/1748-5908-4-42 (PMC2724481; doi:10.1186/1748-5908-4-42)
Supplement: Additional file 2 — Table S3. National level contextual factors potentially influencing effectiveness of the hospital based intervention programme to improve quality of paediatric and newborn care. The data provided indicate how the national health policy context changed during the progress of the study. [file 1748-5908-4-42-S2.doc]

**Table 3**. National level contextual factors potentially influencing effectiveness of the hospital based intervention programme to improve quality of paediatric and newborn care.

| **Baseline**  **Early 2006** | **Baseline-Survey 2**  **August 2006 – February 2007** | **Survey 2 - Survey 3**  **March 2007 – September 2007** | **Survey 3 - Survey 4**  **October 2007 – March 2008** |
| --- | --- | --- | --- |
| Government announces plans to hire nationally 1403 nurses, 144 Clinical Officers & 36 Pharmacy Technicians  Civil servants (including health workers) agree to performance contracts  Enrolled nurse grade under threat, increasing number of nurses taking in-service 'up-grading' courses that reduce their availability to perform routine duties  Transition to a 'pull' system for hospital supplies: hospitals order within a fixed facility budget what they require from the Kenya Medical Supplies Agency (KEMSA). | Performance improvement through the Rapid Results Initiative started at hospital level  Nationwide distribution of new, combination therapy for treatment of malaria and training of 6,800 health workers in new malaria treatment guidelines  Leave allowance and uniform allowance implemented for nurses  Ministry of Health agrees to absorb increased number (100%) of medical graduates entering internship  Government announces 3.5bn KSH ($55million) funding increase for health, with public hospitals identified as major recipients | Government announce new scheme of service for health workers and implement leave allowance and increased salary from July 2007  Director of Medical Services issues circular to all hospitals demanding an improvement in documentation for and quality of newborn care  Director Medical Services issues circular endorsing universal Provider Initiated Counselling and Testing for HIV in hospitals  Ministry of Health requests hospitals to implement a Service Charter  Government formally scraps fees payable for delivery care in public hospitals | First round of performance appraisal for doctors, clinical officers and nurses  Minister for Health sacked, November 2007  National elections take place in December 2007  Post-election violence - specific difficulties with delivery of pharmaceutical and non-pharmaceutical supplies to some hospitals |
|  |  |
