# Supplementary material for: An intervention to improve paediatric and newborn care in Kenyan district hospitals: Understanding the context
Source: Implement Sci. 2009 Jul 23;4:42. doi: 10.1186/1748-5908-4-42 (PMC2724481; doi:10.1186/1748-5908-4-42)
Supplement: Additional file 3 — Table S4. Hospital level contextual factors potentially influencing effectiveness of the hospital based intervention programme to improve quality of paediatric and newborn care. The data provided indicate how the local health policy and organizational context changed during the progress of the study. [file 1748-5908-4-42-S3.doc]

Table 4. Hospital level contextual factors potentially influencing effectiveness of the hospital based intervention programme to improve quality of paediatric and newborn care.

|  | **Baseline**  **Early 2006** | **Baseline-Survey 2**  **August 2006 – February 2007** | **Survey 2 - Survey 3**  **March 2007 – September 2007** | **Survey 3 - Survey 4**  **October 2007 – March 2008** |
| --- | --- | --- | --- | --- |
| **H1** | Hospital receiving assistance from MSF-France for the provision of HIV care  Approximate monthly user fee income $10-13,000 | Senior Nurse in charge of Maternal and Child Health clinic changes  Approximately 25% of hospital nursing staff rotated to new departments  Three new medical officers posted to hospital  PLAN International donate an oxygen concentrator  Hospital management establishes task force to improve revenue collection | Total nursing compliment for hospital falls by 7  Two new Medical Officers posted to hospital, one former MO leaves  Two of 4 hospital nutritionists leave  MSF-France begins to supply ready to use therapeutic foods for children with HIV and severe malnutrition  An NGO (NARESA) donates some new hospital beds | Senior Nurse in charge of Maternal and Child Health clinic changes  Senior Nurse in charge of paediatric ward changes  Vacant Paediatrician post filled 1st October but person leaves before the end of December, a replacement Paediatrician is posted at the start of March 2008  Total nursing compliment falls by 6  Three medical officers left the hospital but eight medical officer interns posted in February 2008  Hospital increases charge for a night in hospital for children from 20 to 50 KSH (US$0.3 to $0.8) |
|  |
|  |  |  |  |  |
| **H2** | Hospital receiving assistance from European Union to provide HIV care  Approximate monthly user fee income $24,000 | Senior Nurse in charge of paediatric ward changes  OPD nurse i/c changed  Approximately 10% of hospital nursing staff rotated to new departments  Renovation of walk-ways, laundry , paediatric ward and outpatient department with government funds  Medical records department built with European Union funds | Hospital Administrative Officer replaced  Clinical Officer in charge of the hospital is changed  Senior Nurse in charge of Maternal and Child Health clinic changes  One of four hospital nutritionists leaves  Renovation of maternity unit and nursery with government and hospital funds  Hospital kitchen renovated with Constituency Development Funds | Rain water tanks to improve hospital water supply are constructed with Constituency Development Funds  Vacant Paediatrician post filled from February 2008  Funding from HIV project partner, Ministry of Health and hospital to support youth friendly HIV services  Delivery of resuscitation couch, 2 incubators, 2 newborn resuscitaire's from government supply |
|  |
|  |
|  |
|  |
|  |
|  |
|  |  |  |  |  |
| **H3** | Hospital receiving assistance from an NGO (NARESA) and Centres for Disease Control to provide HIV care  Approximate monthly user fee income $35-40,000 | Senior Nurse in charge of Maternal and Child Health clinic changes  Approximately 20% of hospital nursing staff rotated to new departments  Renovations of Maternal and Child Health Clinic, Outpatient Department ongoing and new Blood Bank constructed by National Blood Transfusion Service  Hospital begins construction of three new wards to house 150 beds  Hospital wide increase in user fees | Senior Nurse in charge of Maternal and Child Health clinic changes  Senior Nurse in charge of outpatient department changes  Senior Nurse and Medical Officer in charge of paediatric ward change  Seven new nurses posted to hospital  Approximately 25% of Maternal and Child Health and outpatient clinic nurses rotate to new departments  Renovations of outpatient and Pharmacy completed  Initiative to improve PMTCT by NARESA | Senior Nurse in charge of paediatric ward changes  Second paediatrician posted to hospital from October to December 2007 but then posted elsewhere  Total nursing compliment falls by 10  One of three hospital pharmacists leaves  Renovations of maternal and child health clinic completed  UNICEF donate Bag Valve Mask devices |
|  |
|  |
|  |
|  |
|  |
|  |
|  |  |  |  |  |
| **H4** | Hospital receiving assistance from German government through a voucher payment for delivery care 5,000/= normal delivery, 21,000/= for CS  Approximate monthly user fee income $10,000 | Nursing Officer in charge of the hospital retires  Two of five Medical Officers leave  Senior Nurse in charge of maternity ward changes  Ward renovations underway with relocation of patients | Hospital Administrative Officer replaced  One of three Medical Officers leaves, new Obstetrician posted  Approximately 25% of Clinical Officers rotated in district with 25% of hospital compliment new to hospital  Elizabeth Glaser Foundation supporting PMTCT | New Nursing Officer in charge of the hospital appointed  One of two Medical Officers leaves, two of three nutritionists leave and seven nurses leave  Eye unit completed with support from Rotary International  Delivery of 2 neonatal incubators, an autoclave and laundry machine from government supply |
|  |
|  |
|  |
|  |  |  |  |  |
| **H5** | Hospital receiving assistance from a Kenyan programme (AMPATH) and MSF-Spain to provide HIV care  Approximate monthly user fee income $10-13,000 | Hospital repainted and minor renovations made with government funds  New Comprehensive Care Clinic for HIV services constructed by partners  Drug procurement switched to government supplier at the close of a donor funded project  Approximately 90% of hospital nursing staff rotated to new departments | Hospital Medical Superintendent changed  New obstetrician posted to hospital  Paediatric ward toilets rehabilitated  Bore hole to provide water established and sponsored by local NGO and Church groups | Hospital Medical Superintendent changed  Ten nurses left and not replaced  Hospital reviews and increases all user fees |
|  |
|  |
|  |
|  |
|  |
|  |
|  |
|  |  |  |  |  |
| **H6** | UNICEF and Save the Children (Kenya) provide assistance for treatment of malnutrition including ready to use therapeutic feeds (F75 and F100)  Elizabeth Glaser Foundation and a local programme (APPHIA II) provide support for HIV services  Approximate monthly user fee income $10,000 | Renovation of paediatric wards, maternity and the outpatient department undertaken with government and hospital funds | Hospital Medical Superintendent changed  Hospital Administrative Officer replaced  One Medical Officer posted allowing allocation of a doctor to the paediatric ward  Four Clinical Officers and one Pharmacist posted  Bore hole to provide water established and sponsored by APPHIA II | Hospital Medical Superintendent changed  Hospital Administrative Officer replaced (again)    Nursing Officer in charge of the hospital changed  Four clinical officers and one Pharmacist leave  Vacant Obstetrician post filled |
|  |
|  |
|  |
|  |
|  |
|  |  |  |  |  |
| **H7** | A local programme (APPHIA II) provides support for HIV services  Approximate monthly user fee income $30-32,000 | Renovation of adult wards, and the outpatient department undertaken with government and hospital funds  Second borehole to provide water established with hospital funds | Ten new nurses posted to hospital  Senior Nurse in charge of outpatient department changes  Renovation of the paediatric ward and hospital kitchen with government and hospital funds  Delivery of 6 new incubators and newborn Bag Valve Mask devices  APHIA II renovated Comprehensive Care Clinic for HIV services  Japanese government to provide assistance to Kenyan government to improve hospital | Nineteen nurses left and not replaced  Second paediatrician posted |
|  |
|  |
|  |
|  |
|  |
|  |
|  |
|  |  |  |  |  |
| **H8** | Hospital receiving assistance from an HIV project to provide HIV services and from the European Union providing computers and beds  Approximate monthly user fee income $18-20,000 | Approximately 10% of hospital nursing staff rotated to new departments | Two new Medical Officers posted to hospital and two new Pharmacists  Renovation of toilet facilities in outpatient department  Construction of a new operating theatre with hospital funds begins | Construction of a new comprehensive care clinic for HIV services begins  Hospital decides not to accept Clinical Officer Interns for supervised work experience |
